# Peptide Markers for Rapid Detection of KPC Carbapenemase by LC-MS/MS

**DOI:** 10.1038/s41598-017-02749-2

**Published:** 2017-05-31

**Authors:** Honghui Wang, Steven K. Drake, Jung-Ho Youn, Avi Z. Rosenberg, Yong Chen, Marjan Gucek, Anthony F. Suffredini, John P. Dekker

**Affiliations:** 1 Critical Care Medicine Department, Clinical Center, National Institutes of Health, Bethesda, Maryland USA; 2 Microbiology Service, Department of Laboratory Medicine, Clinical Center, National Institutes of Health, Bethesda, Maryland USA; 3 Kidney Disease Section, National Institute of Diabetes and Digestive and Kidney Diseases, National Institutes of Health, Bethesda, Maryland USA; 4 Department of Pathology, Johns Hopkins University, Baltimore, Maryland USA; 5 Proteomics Core Facility, National Heart, Lung and Blood Institute, National Institutes of Health, Bethesda, Maryland USA

## Abstract

Carbapenemase producing organisms (CPOs) represent an urgent public health threat, and the need for new rapid methods to detect these organisms has been widely recognized. CPOs carrying the *Klebsiella pneumoniae* carbapenemase (*bla*_*KPC*_) gene have caused outbreaks globally with substantial attributable mortality. Here we describe the validation of a rapid MS method for the direct detection of unique tryptic peptides of the KPC protein in clinical bacterial isolates with an isolate-to-result time of less than 90 minutes. Using a genoproteomic discovery approach that combines theoretical peptidome analysis and liquid chromatography-tandem MS (LC-MS/MS), we selected three high abundance peptide markers of the KPC protein that can be robustly detected following rapid tryptic digestion. Protein BLAST analysis confirmed that the chosen peptide markers were unique to KPC. A blinded validation set containing 20 KPC-positive and 80 KPC-negative clinical isolates, performed in triplicate (300 runs) demonstrated 100% sensitivity and 100% specificity (60/60 positive identifications, 240/240 negative identifications) using defined rules for positive calls. The most robust tryptic peptide marker in the validation was LTLGSALAAPQR. The peptide discovery and detection methods validated here are general and should be broadly applicable to allow the direct and rapid detection of other resistance determinants.

## Introduction

Carbapenemase producing organisms (CPOs) that are resistant to carbapenems and to most or all other antibiotics have been recognized as an increasingly urgent threat to global public health^1, 2^. CPOs carrying the *Klebsiella pneumoniae* carbapenemase gene (*bla*_*KPC*_) have caused hospital outbreaks globally, and isolates collected from these outbreaks have been analyzed extensively with genomic methods^3–9^. The 2015 White House National Action Plan for Combating Antibiotic-Resistant Bacteria recognized as one of its major goals the need for new rapid detection methods for highly resistant gram-negative organisms, such as KPC-producing isolates^10^. Given the prevalence of KPC-mediated carbapenem resistance and the diversity of species in which this plasmid-borne gene is now found, new methods for rapid detection of KPC are paramount.

A number of rapid mass spectrometry (MS) methods are available for detecting carbapenem hydrolysis activity in cultured isolates^11–21^, and a rapid method for detecting a proxy protein genetically-linked to *bla*_*KPC*_ in certain plasmids has been developed^22, 23^. However, a potential limitation of these approaches is that they are indirect. A more direct approach that uses MS to detect tryptic peptides of carbapenemases has been suggested^24^, and a variety of related methods have been studied^25^. A shotgun proteomics-based method using microwave-assisted tryptic digestion followed by liquid chromatography (LC)-nano-electrospray ionization trap MS identified OXA-family beta-lactamases and the carbapenem-resistance associated CarO protein from *Acinetobacter baumannii* ^26^, and a method based on capillary electrophoresis-electrospray ionization(CE-ESI) MS/MS was able to identify tryptic peptides of KPC and OXA-48 carbapenemases from cultured isolates reliably for accurate classification^27^. These studies have demonstrated the potential power of these approaches, but the reported turnaround times of 5–16 hours are not optimally suited for the diagnostics workflow in the clinical microbiology laboratory. Recently, a method based on ESI-triple quadrupole MS (ESI-QqQ MS) and rapid selected reaction monitoring (SRM) detected tryptic peptides of PBP2a and PBP2c in methicillin-resistant *S. aureus* in 60–80 minutes^28^, demonstrating the in-principle applicability of such methods to the diagnostics time scale in a clinical microbiology laboratory.

Here we validate a rapid MS method for the direct detection of unique tryptic peptides of the KPC protein in clinical isolates with an isolate-to-result time of less than 90 minutes. To select high-abundance, specific peptide markers, we applied a genoproteomic discovery approach that combines theoretical genomics-based peptidome analysis with rapid, thermal-assisted digestion and LC-MS/MS^29^. Using this method, we selected three high-abundance peptide markers that are unique to the KPC protein and can be robustly identified following rapid tryptic digestion. The peptide discovery, validation, and detection methods we employ here are general and should be broadly applicable to the direct detection of other components of the bacterial resistome using mass spectrometry.

## Results

### Genoproteomic approach defines theoretical peptide markers

**Figure 1:**
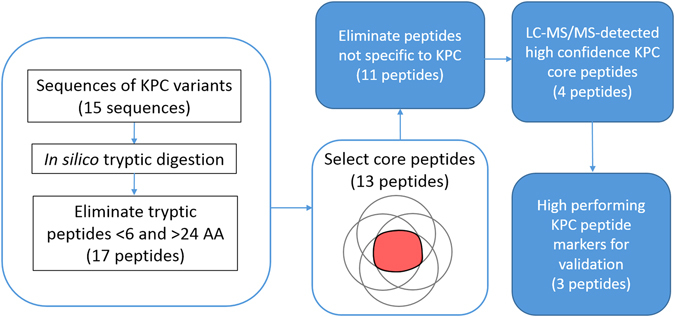
Genoproteomic approach to identify and detect high performing core peptides specific to the KPC protein. A multiple sequence alignment of 15 KPC variants was subjected to *in silico* tryptic digestion. Peptides < 6 and > 24 amino acids in length were eliminated, and a set of core peptides common to at least 14 KPC variants was selected. Analysis with BLASTP and LCA were used to eliminate peptides not specific to KPC. High performing peptides were selected for validation from the set of high confidence core peptides that could be detected by LC-MS/MS. The numbers of peptides in parentheses are the numbers remaining after each selection step.

Figure 1 shows the design of the genoproteomic approach used to detect high abundance, specific core (common) peptides of the KPC protein. Starting with an alignment of 15 KPC variants (Supplementary Figure 1), we identified 13 core tryptic peptides for KPC, after excluding peptides with lengths < 6 AA and > 24 AA (Supplementary Table 1). To eliminate those core peptides that were not specific for KPC, lowest common ancestor (LCA) (http://unipept.ugent.be/) and protein BLAST search (https://blast.ncbi.nlm.nih.gov/Blast) were used. The remaining core peptides specific to KPC (n = 11) were chosen as candidate peptide markers for further study.

### Experimental detection of theoretically-determined peptide markers

Among the theoretically-determined core peptides specific to KPC, we sought to identify “high responding peptides”; peptides that were highly abundant and were efficiently ionized thus making them more readily detectable by LC-MS/MS^30^. For experimental method development, we chose two *E. cloacae* complex (ECNIH2 and ECNIH3) and two *K. pneumoniae* isolates (KPNIH1 and KPNIH10) that had been previously sequenced and were known KPC-producers (Table 1)^3, 4^. Supplementary Table 2 lists the number of total proteins and total peptides including the number of core peptides specific to KPC detected by LC-MS/MS in protein extracts from each isolate. The spectral counts for each of the 13 core peptides in 4 isolates are listed in detail in Supplementary Table 1. Four detected KPC-specific core peptides were chosen as potential peptide markers for KPC after the following eliminations. Three core peptides (DTSSPR, FPLCSSFK, and GNTTGNHR) were not detected by LC-MS/MS and were eliminated from further study. Peptides AVTESLQK and SIGDTTFR were frequently detected but were determined not to be specific to KPC by BLASTP and also eliminated. AAVPADWAVGDK and QQFVDWLK were eliminated due to their missed detection in isolate ECNIH2. Both SQQQAGLLDTPIR and ELGGPAGLTAFMR were eliminated due to either incomplete digestion or oxidation. The precursor ions and MRM transitions of the remaining four peptide markers selected for further study are listed in Table 2.

**Table 1:** KPC+ and KPC− isolates used in assay development and validation.

Name	Assay development		Validation	
	KPC−	KPC+	KPC−	KPC +
*Achromobacter sp*.			2	
*Acinetobacter baumannii complex*			1	
*Acinetobacter sp*.			1	
*C. freundii complex*			5	2
*C. koseri*			2	
*E. aerogenes*				2
*E. cloacae complex*		ECNIH2, ECNIH3	8	3
*E. coli*	1		23	1
*K. oxytoca*			3	1
*K. oxytoca/Raoutella ornitholytica*			1	
*K. pneumoniae*	1	KPNIH1, KPNIH10	13	9
*P. aeruginosa*			12	
*P. mirabilis*			1	
*Pantoea sp*.			1	2
*Rhizobium radiobacter*			1	
*S. maltophilia*			3	
*Serratia liquifaciens*			1	
*Serratia marcescens*			1	
*Sphingomonas sp*.			1	
Total	2	4	80	20

**Table 2:** Four high-confidence core peptide markers for KPC.

Peptide	Charge	Precursor	Collision Energy	T1	T2	T3	T4	T5	T6	T7
APIVLAVYTR	2+	551.83	19	722.41 (y6)	821.48 (y7)	609.33 (y5)	934.57 (y8)	538.29 (y4)		
LTLGSALAAPQR	2+	599.35	17	542.30 (y5)	400.23 (y3)	471.26 (y4)	870.47 (y9)	655.38 (y6)	726.42 (y7)	983.56 (y10)
GFLAAAVLAR	2+	494.80	12	671.41 (y7)	600.38 (y6)	529.34 (y5)	784.50 (y8)			
LALEGLGVNGQ^a^	2+	535.79	11	597.36 (b6)	753.45 (b8)	427.25 (b4)	484.27 (b5)	867.49 (b9)		

^a^Only b-ions were included in data processing due to deamidation in N or Q even though all b-ion and y-ion data were available. This peptide was not included in the validation set due to deamindation.

### Optimization of rapid tryptic digest protocol

To simplify sample processing and to optimize the rapid digestion protocol, we used a targeted LC-MS/MS method on a Q-TOF to compare the relative signal intensities of the four candidate peptide markers with various sample processing and digestion protocols. This approach allowed us to evaluate the consequences of protocol modification directly on the KPC peptides of interest. To achieve better digestion efficiency with fewer missed cleavages in a short digestion time, we chose Trypsin/Lys-C mix for digestion, which was reported to improve the digestion by reducing missed lysine cleavages and improving signal intensity for some peptides for accurate absolute protein quantification^31^. We compared the intensities of the 4 potential peptide markers with one KPC positive and one KPC negative isolate. Supplementary Figure 2 shows digestion protocol optimization. 1 ug enzyme was used to digest 20 uL lysate in all tests. In these experiments, microwave treatment did not enhance the digestion efficiency compared with water bath, consistent with previous results^29^. Trypsin/Lys-C also did not improve the detection of these 4 KPC peptides compared with Trypsin only, and longer digestion did not produce significantly higher signals on the targeted peptides. While a more complete tryptic digestion could be achieved with a longer digestion time (e.g. 4 hour or overnight), our objective in this work was to optimize the digestion protocol so that it yielded quantities of the chosen peptide markers that were sufficient for robust determination of the presence or absence of KPC protein in the shortest time, to be compatible with a clinical assay. Thus, 15 min digestion at 55 °C in water bath was chosen for all clinical isolates and was used as the standard method for the blinded validation (below).

### Evaluation of method performance

We tested four known *bla*_*KPC*_ positive isolates (ECNIH2, ECNIH3, KPNIH1, KPNIH10) and two *bla*_*KPC*_ negative isolates to study reproducibility, background signals, carryover effect, interfering peaks, and peptide stability. An in-line external standard (external positive control), composed of protein extract made from a KPC-expressing isolate (KPNIH1, as described in Materials and Methods), was tested at the beginning and end of each day, to ensure consistent instrument performance and to allow a quantitative measure of instrument drift from the beginning to the end of the testing day (20 hour period). Protein extract from a KPC-expressing bacterial isolate was chosen as opposed to purified or labeled peptides to replicate as closely as possible the complex protein matrix of the test samples. In the blinded validation (below), this external control was run before and after each set of 30 protein extractions, and the average intensity value for each peptide in the external control between these two control points served as a reference for calculations. As the purpose of the assay was to determine the presence or absence of KPC protein, and not quantification of amount, an internal peptide standard was not included.

To assess specimen-to-specimen reproducibility of peptide signal intensity and background, we calculated the intensity ratio of each detected peptide from each measurement relative to the averaged intensity of the two daily external positive control runs for ECNIH2, ECNIH3, KPNIH1, KPNIH10 (all *bla*_*KPC*_ positive). Results for these measurements performed in technical triplicate are presented in Table 3. For LTLGSALAAPQR, the range of intensity ratios for four *bla*_*KPC*_ positive isolates (12 measurements) was 0.61 to 2.31, while the intensity ratios for two *bla*_*KPC*_ negative isolates were all < 0.01. Though moderate variation in relative intensity was found, positive isolates were robustly and reproducibly separated from negative isolates, and overall background was low. A signal-to-noise ratio was calculated representing the lowest positive value to the highest negative value (Table 3), and these calculations confirm that the relative magnitude of fluctuations in absolute signal intensity were very small relative to the signal-to-noise ratio. Among the four potential peptide markers, LTLGSALAAPQR has the highest ratio and may be the best single peptide marker for KPC.

**Table 3:** Intensity ratios for 4 peptide markers for 6 test development set isolates.

Isolate	KPC	Sample set	GFLAAAVLAR	LTLGSALAAPQR	APIVLAVYTR	LALEGLGVNGQ^a^
ECNIH2	+	A	3.47	2.16	2.89	1.35
ECNIH2	+	B	3.06	1.67	2.61	1.12
ECNIH2	+	C	3.09	1.69	2.52	1.13
ECNIH3	+	A	1.11	0.70	1.03	0.54
ECNIH3	+	B	1.24	0.65	0.99	0.49
ECNIH3	+	C	1.14	0.61	0.91	0.48
*E. coli*	*−*	A	0.03	0.00	0.02	0.11
*E. coli*	*−*	B	0.02	0.00	0.01	0.10
*E. coli*	*−*	C	0.02	0.00	0.02	0.11
*K. pneumoniae*	*−*	A	0.03	0.00	0.05	0.03
*K. pneumoniae*	*−*	B	0.03	0.00	0.05	0.03
*K. pneumoniae*	*−*	C	0.04	0.00	0.04	0.03
KPNIH1	+	A	2.62	2.31	2.80	1.81
KPNIH1	+	B	2.77	1.44	2.23	1.25
KPNIH1	+	C	3.26	1.64	2.54	1.29
KPNIH10	+	A	3.15	2.30	2.63	1.69
KPNIH10	+	B	3.06	1.84	2.75	1.38
KPNIH10	+	C	2.10	1.33	1.67	1.37
Positive range			1.11–3.47	0.61–2.31	0.91–2.89	0.48–1.81
Negative range			0.02–0.04	<0.01	0.01–0.05	0.03–0.11
Lowest Pos/Neg ratio			27.8	>61.0	18.2	4.4

Intensity ratio is calculated as signal peak intensity/daily positive control intensity. Technical triplicate samples (A, B, and C) were prepared at the same time. ^a^Based on b-ions only due to N or Q deamination; this peptide was not used in validation.

No detectable carryover was observed for 3 of the KPC peptides. However, for the peptide GFLAAVLAR nominal carryover signals were detected in *bla*_*KPC*_ negative isolates (Supplementary Figure 3). During test development, carryover was distinguished from true signal by subsequent measurement with a blank. While the carryover signal may have the correct retention time and transition rank order, the intensity is weak and the signal disappears on repeat testing after further column cleaning. In cases deemed to be due to carryover, the GFLAAAVLAR signal was observed immediately after a sample with a large positive signal, and accounted for only 1–2% of the signal detected from previous positive specimens. These values were well below the signal-to-noise ratios of almost all observed true positives.

Weak interfering peaks were observed for APIVLAVYTR as shown in Supplementary Figure 4. Interfering peaks were defined as those detected signals from non-target peptides that had similar total mass and retention time to the intended target peptide. The interfering peaks for APIVLAVYTR were easily distinguished by comparison of their mass spectra with the intended targets.

While most of the studied peptides were found to be stable, deamidation of the LALEGLGVNGQ peptide was observed during the test run, and the signal for this peptide decreased with time while the sample was in the autosampler at room temperature (Supplementary Figure 5). While it was possible to monitor b-ions for both deamidated and native peptides (data not shown), this peptide was not analyzed in the validation set described below (Supplementary Material, Note 1).

### Blinded validation set

To test our method, we constructed a validation set of 100 de-identified clinical isolates run in triplicate, consisting of 20 KPC-positive and 80 KPC-negative isolates (Table 1). All 300 runs were treated independently with replicates randomly interleaved and unlinked. Testing, consisting of running digested protein extracts on the LC-MS/MS instrument and interpreting resulting spectra, was performed by a single expert operator who was blinded to the identity of the isolates and to the number of KPC positives and negatives in the set. The operator submitted the full list of determinations for 300 measurements prior to unblinding, and the list was matched to the isolate *bla*_*KPC*_ PCR results by an independent second analyst.

To score an isolate in the validation set as KPC positive or negative, the operator examined two factors: (1) The MS/MS spectral transition rank order in conjunction with the retention time for each of the three tryptic peptides. (2) The ratio of signal intensity to that of the daily positive control for each of the three tryptic peptides. If the intensity ratio for a given peptide was < 0.10, it was scored as negative. If the ratio was > 0.10, the signal was manually examined and the alternatives considered were true signal, carryover (for GFLAAAVLAR only), interfering signal, and noise. Carryover was ruled out for GFLAAAVLAR if the preceding sample was negative. If the preceding sample had a positive GFLAAAVLAR signal (indicating the possibility of carryover), then the other two peptides were evaluated. If both other peptides were ruled negative, then the > 0.10 intensity GFLAAAVLAR signal was attributed to carryover, and the sample was called negative. If at least one of the other two peptides was ruled as positive, the > 0.10 intensity GFLAAAVLAR signal was also ruled as positive. All three peptides were evaluated to exclude interfering peptides and noise based on transition rank orders and retention time, as characterized during method development. For a sample to be called positive, two of three evaluated peptides had to be scored as positive.

Supplementary Table 3 lists the measured averaged intensities for three peptide markers for the positive control during the 10-day testing period and the percentage change in intensity for each peptide over the course of each day of testing. This percentage represents the relative change due to instrument drift during the testing day (~20 hours). The absolute peak intensities for each peptide marker varied during the 10-day period and should not be used for absolute comparison. Importantly, the variations in intensities over the course of the testing day were found to be insignificant relative to the signal-to-noise ratios, and did not impact the ability to determine presence or absence of each peptide marker.

### Validation set performance

**Figure 2:**
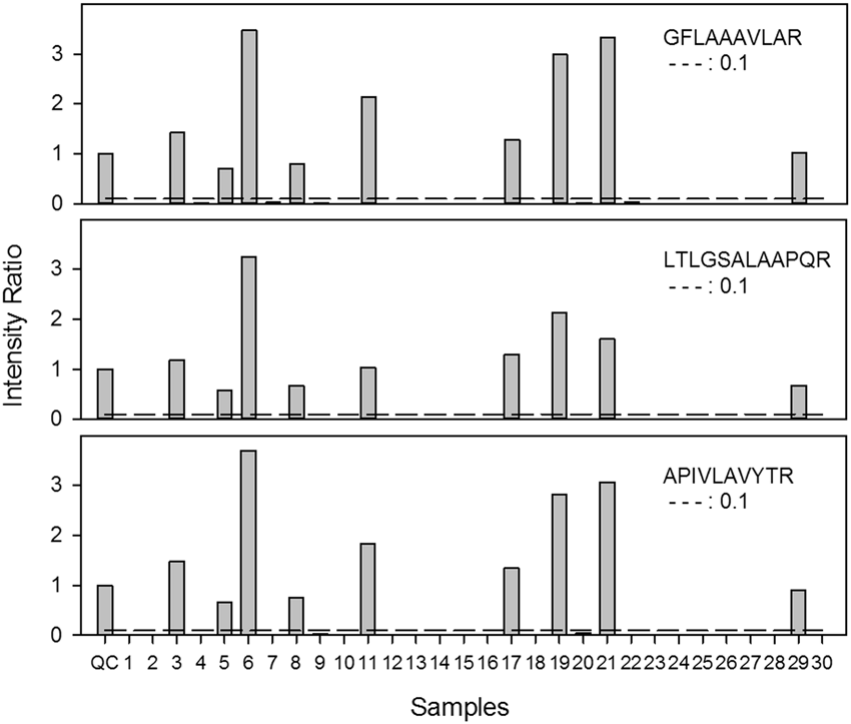
Relative intensities of the three selected KPC peptide markers shown for consecutive validation samples #1–30. The intensity was normalized to daily positive controls, tested as the first and last sample of each testing day. Samples 3, 5, 6, 8, 11, 17, 19, 21, and 29 were called positive in validation and confirmed to be KPC positive by PCR.

**Figure 3:**
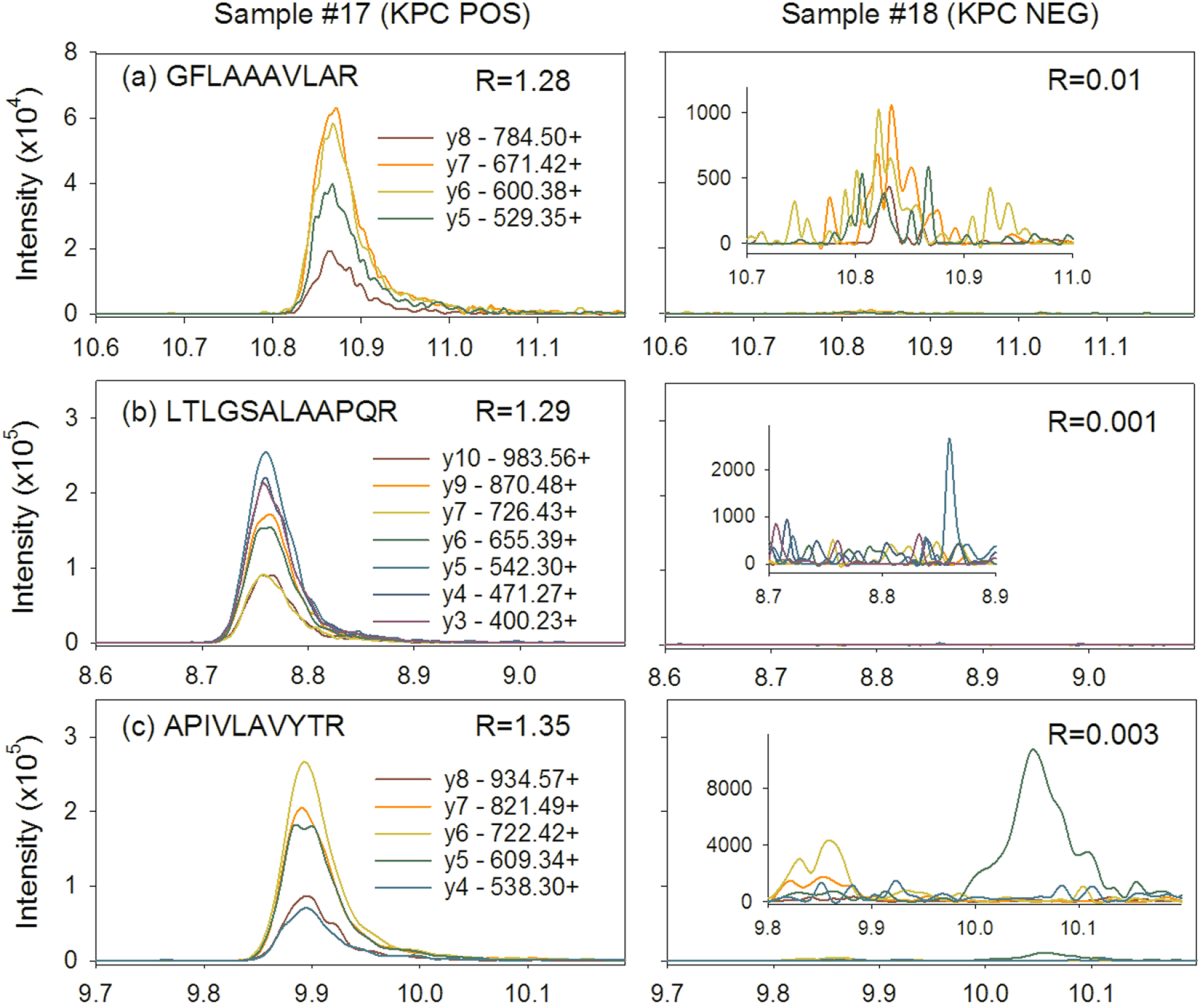
The LC-MS/MS chromatograms of the three chosen peptide markers are shown for two consecutive validation test set samples to demonstrate positive signals, noise, and carryover effects: (**a**) is GFLAAAVLAR, (**b**) is LTLGSALAAPQR, and (**c**) is APIVLAVYTR. Sample #17 is KPC-positive, and #18 is KPC-negative. The outside axes are drawn to the same scale in all figures. Given the very high positive-to-negative signal ratio, an inset is included with a zoomed-in vertical scale for sample #18. Normalized Intensity ratios for the three peptides in specimen #17 (KPC-positive) ranged from 1.28–1.35. Normalized intensity ratios for the three peptides in specimen #18 (KPC-negative) ranged from 0.003–0.01.

Blinded testing by an expert operator correctly identified all 20 KPC positive isolates in unlinked triplicate samples (60/60 correct identifications). No false positives occurred among the 80 KPC negative isolates tested (240/240 correct negative calls), yielding an overall performance of 100% sensitivity and 100% specificity for detection of KPC protein in the blinded test set. Figure 2 shows the relative intensities of the three KPC peptide markers among the first 30 consecutive test samples and is representative of the signal to noise for the rest of the samples. The relative intensities of all KPC peptides (calculated as a ratio to the average positive control value for that day of testing) ranged from 0.18 to 14.81. Figure 3 shows the representative chromatograms of three peptide markers from one *bla*_*KPC*_-positive and one comparator *bla*_*K*PC_-negative clinical sample that were run sequentially, to illustrate the relative sizes of positive signals, noise, and carryover effects.

### Assessment of dynamic range of targeted LC-MS/MS

We performed an analysis using serial dilutions to assess the dynamic range of the targeted LC-MS/MS assay relative to the starting extract concentration prepared by the described method. The lysate of KPNIH1 was spiked into the lysate of a *bla*_*KPC*_-negative *K. pneumoniae* isolate (included to simulate the matrix effect) to a final volume of 20 μL. The averaged intensity ratios based on triplicate experiment are shown in Supplementary Table 4. The measured ratios correlated well with the theoretical ratios, indicating linearity of the assay over this tested range. LTLGSALAAPQR and APIVLAVYTR were found to have the highest dynamic ranges. The spectral MS/MS transition rank orders for LTLGSALAAPQR can still be observed through an 80-fold dilution (Supplementary Figure 6). However, we would note that signals with intensity below 0.10 would be called negative by the expert rules applied to our validation set, and relative intensities dropped below 0.10 after an 8-fold dilution factor.

## Discussion

We sought to validate a rapid, MS-based method to identify the KPC carbapenemase protein directly in cultured clinical isolates. Using a genoproteomic approach that combines theoretical peptidome analysis with experimental LC-MS/MS^29^, we selected three high abundance and specific tryptic peptides of the KPC protein that could be robustly detected by LC-MS/MS following rapid tryptic digestion. To characterize the performance of this method, we constructed a validation set from 20 *bla*_*KPC*_-positive and 80 *bla*_*KPC*_-negative clinical isolates. Though in many hospitals in the United States, *bla*_*KPC*_ occurs in fewer than 1% of all Gram-negative clinical isolates, we felt that a validation set reflecting this natural frequency would not adequately challenge the method, justifying inclusion of a larger fraction of positive isolates. Each isolate was tested in randomly-interleaved triplicate (300 runs total) by one blinded expert operator. Using rule-based calls developed from the study of sources of interference and variability, single operator evaluation of the test set demonstrated 100% sensitivity and 100% specificity (60/60 positive identifications, 240/240 negative identifications).

Our method relied on the development of a rapid tryptic protein digestion technique. In previous work, we and others have shown that thermal-assisted digestion demonstrated equivalent performance to microwave-assisted digestion^29, 32^. In the current study, we chose tryptic digestion for 15 minutes at 55 °C. This procedure is rapid, simple, and requires only a water bath and a vortexer in a 50 °C incubator. Performed on a FA/ACN lysate identical to that used for many routine MALDI-TOF MS instruments, our rapid method yielded a sufficient concentration of KPC-specific peptide to be robustly detected by LC-MS/MS.

Several technical issues in this study required careful consideration. The interpretation of results required rule-based evaluation of the MS/MS spectra by an expert operator familiar with the analysis of interfering signals and instrument behavior. Distinguishing interfering peptides from target peptides in unknown clinical isolates also required detailed comparison of MS/MS transition rank orders, individual transition spectra, and retention times. As an example, we found that one *bla*_*KPC*_-negative *K. pneumoniae* isolate yielded two transition peaks that resembled the y6 and y7 fragments for APIVLAVYTR as shown in Supplementary Figure 4. These two peaks demonstrated a retention time of 9.9 min and were found to represent fragments whose monoisotopic m/z values were approximately 1 Da less than those of the monoisotopic peaks of y6 or y7 of APIVLAVYTR from KPC. Consequently, the observed y6-similar ion was from a fragment with m/z value of y6-1, and its first isotopic peak overlapped with the y6 monoisotopic peak. The same was true for y7 ion. However, in this and other such cases, examination of the transition rank orders distinguished interfering peptides unambiguously from the three target peptides. Thus, spectral examination was necessary for evaluation, and, with weak signals, distinguishing true peaks from interfering peaks may be difficult.

In choosing the best MS approach that is suitable to routine laboratory workflow and that optimizes sensitivity and specificity, both mass accuracy and instrument analysis time must be taken into account. A total proteomics approach may reduce risks of false positives posed by interfering peptides, but such an approach requires a longer gradient time, increasing the time-to-result. Targeted LC-MS/MS using high mass accuracy Q-TOF, on the other hand, provides a more rapid approach to peptide assay development without labeled peptides. High mass accuracy spectrometers have advantages in distinguishing false positives from interfering peptides with a similar mass. For clinical testing, a workflow based on multiple reaction monitoring (MRM) using a QQQ instrument may improve speed and sensitivity, but the lower mass resolution may limit the recognition of problematic interfering near-mass peaks.

Several factors may contribute to the quantitative differences observed in the KPC core peptides detected using the methods described. First, incomplete digestion may occur with the rapid approach. This is an expected consequence of a method that was designed to find easily detectable peptides rather than providing comprehensive sequence coverage. Second, MS detection efficiency was not equivalent for each of the resulting tryptic peptides. A third factor relates to differences in ionization efficiency or completeness of MS/MS fragmentation. For instance, it is known that some peptides may undergo in-source fragmentation, while others may not^33^. Further, the existence of multiple charged ions for the same peptide may also affect the detection sensitivity (e.g. double and triple charged ions of LALEGLGVNGQ were observed). The ratio of double/triple charged ions is affected by the instrument settings, the choice of mobile phases, and the water source. The use of labeled peptides in MRM can correct these types of variation for absolute quantification, but in the current study using the targeted LC-MS/MS assay by Q-TOF without labeled peptides, absolute quantification was not necessary and the peptides that produce the highest ion-current response (high-responding peptides) appear to provide the best detection sensitivity^30^.

The relative ratio intensities for 60 KPC-positive isolate measurements ranged from 0.13 to 4.45 for peptide marker LTLGSALAAPQR, indicating that there was up to a 34-fold variation in the relative concentration of the detected LTLGSALAAPQR among the tested clinical isolates. We are unable to attribute these differences in detected peptide concentrations to differences in KPC expression in the isolates, as we did not quantify the number of cells used for cell lysis or account for differential lysis efficiency. This variation in absolute intensities was still small relative to the signal-to-noise ratio and did not significantly impact the ability to determine presence or absence of KPC protein, as demonstrated by the validation set performance.

Spontaneous peptide chemical modifications such as N or Q deamidation, or M oxidation may reduce the detection sensitivity and assay reliability. As noted above, the deamidation of the LALEGLGVNGQ peptide during the course of the testing day resulted in two peaks, which complicated data analysis. Thus, we chose not to use LALEGLGVNGQ for the validation, given the identification of three well-performing peptide markers that were not subject to substantial deamidation. In cases where detection relies on a single peptide that is subject to deamidation, interpretation may be more complicated, but a 4 °C autosampler may help to reduce deamidation.

HPLC-MS carryover also may result in false positives in samples following true positives with strong KPC signals. Such carryover can be influenced by instrument factors (e.g. needle, injector, valves and tubes), frequency and method of column cleaning, and the peptide’s intrinsic chemistry. An 18-min blank run with two cycles of washing/regeneration was inserted between samples to reduce the carryover in our setup. We repeated measurement on one sample (#80) from the validation set that demonstrated GFLAAAVLAR carryover to confirm that the expert rule-based interpretation was correct. In this case, the signal disappeared on repeat testing, confirming that the signal was due to carryover, and not due to some other unexpected source. In all cases in this study, possible carryover was recognized easily by the signal intensity of the preceding sample and the ratio of intensities of true positive and the sample with carryover.

Lastly, retention time shift is another source of potential variability in the selection of peak detection in Q-TOF without labeled peptides. The use of daily positive controls at the beginning and at end of the day of testing was essential to the identification of any retention time shifts that occur within the context of normal operation.

In conclusion, we have validated a rapid MS method for the direct detection of tryptic-digest fragments of the KPC protein in clinical isolates. Using a genoproteomic discovery approach that combines theoretical peptidome analysis with experimental LC-MS/MS, we selected three high abundance and specific peptide markers of the KPC protein that can be robustly identified following rapid (15 minute) tryptic digestion. The most robust tryptic peptide marker was LTLGSALAAPQR. The genoproteomic peptide discovery approach validated here is general and should be broadly applicable to allow the direct and rapid detection of other components of the bacterial resistome using mass spectrometry.

## Materials and Methods

### Bacterial isolates

106 de-identified, subcultured bacterial isolates (Table 1) collected from NIH Clinical Center patients during the course of routine laboratory evaluation were used in this study and included previously published, sequenced isolates where noted by formal names and publication citations^3, 4^. Six of these isolates were used for the test development phase, and 100 were used for the test validation phase. All bacterial isolates were grown on blood agar plates (Remel, Lenexa, KS) for 18 – 24 hr at 35 °C with 5% CO_2_, and lysed with formic acid (FA) and acetonitrile (ACN) as described previously^22^ with minor modifications as described below. Briefly, for each sample, a 10 μL loop of fresh bacterial cells was resuspended in 0.5 mL 70% ethanol, vortexed for 1 min and centrifuged at 20,800 g for 2 min. Supernatant was removed and the pellet was resuspended in 100 μL of 70% FA and mixed to homogeneity, followed by addition of 100 μL of 100% ACN. The resulting solution was re-vortexed for 10 s and centrifuged for 2 min at 20,800 g. 150 μL of supernatant (FA/ACN lysate) was stored at −20 °C for later use. For technical replicates in the validation set, the preceding steps were performed three times from different regions of the same culture plate.

### Confirmation of genus and species

The identity of all isolates used in this study was confirmed by MALDI-TOF MS (Bruker MicroFlex LT mass spectrometer, Bruker Daltonics, Billerica, MA) from the same plates that were used to create protein extractions. Isolates were spotted onto a target plate, overlaid with 2 μL alpha-cyano-4-hydroxycinnamic acid (α-CHCA) and dried prior to analysis^22^.

### Confirmation of presence or absence of bla_KPC_ gene

All isolates in this study (Table 1) underwent PCR to verify the presence or absence of the *bla*_*KPC*_ gene using the CDC protocol^34^. Additionally, all *bla*_*KPC*_ positive isolates used for method development had previously undergone whole genome sequencing, as noted.

### Daily external standard (positive control)

200 μL FA/ACN lysate of a KPC-positive isolate (*K. pneumoniae* KPNIH1) from the test development set was mixed with 200 μL FA/ACN lysate of a KPC-negative *K. pneumoniae* isolate to create a “half-concentration” solution to approximate a middle point of the expected signal. This solution was used as the positive control. 20 μl of the daily positive control solution was treated identically to the blinded validation isolate extracts and was run as the first and last sample of each testing day to confirm assay performance and for the purpose of MS/MS spectral comparisons of KPC peptide markers with unknowns in the validation set.

### Tryptic protein digestion

20 μL of FA/ACN lysate was dried with a DNA Speed Vac (model DNA110, Savant) at high heat setting for 15 min. The intact proteins were re-suspended in 100 μL of 100 mM of NH_4_HCO_3_ and vortexed for 15 min at 50 °C. For targeted LC-MS/MS, rapid digestions were carried out either in water bath or using a CEM Discover microwave system (CEM, Matthews, NC) for 15–60 min at 55 °C as described previously^29^ with addition of 1.0 μg of Trypsin or Trypsin/Lys-C mix (Promega, Madison, WI). Digested samples were spun for 4 min at 12,800 g and 90 μL of supernatant was transferred to a glass vial (Thermo Fisher Scientific, CERT5000993) for LC-MS/MS analysis or was stored at –20 °C for later use. For protein identification by Orbitrap Fusion LC-MS/MS, the trypsin-digested solution was diluted 160-fold with 50 mM NH_4_HCO_3_ and 10 μL of the diluted solution was loaded to LC-MS/MS for total protein identification.

### Protein identification by Orbitrap mass spectrometers

Bottom-up protein identification was carried out on an Orbitrap Fusion mass spectrometer (Thermo Fisher Scientific, San Jose, CA) as previously described^29^. LC-MS/MS data were searched against a custom FASTA database composed of Sprot_Human, KPNIH1, and ECNIH2 FASTA files by Proteome Discoverer 1.4 (Thermo) and Scaffold 4 (Proteome Software Inc., Portland, Oregon) as previously described^22, 29^.

### Targeted LC-MS/MS

Targeted LC-MS/MS was run on an Agilent 6540 Q-TOF with AdvanceBio Peptide Mapping column (2.1 × 150 mm 2.7 μm) as described previously^29^. The mobile phases were 0.1% FA, 2% ACN in H_2_O, and 0.1% FA in ACN. The gradient was run from 10% to 35% B over 10 min with a flow rate of 0.4 ml/min. 40 μL of the digested solution was loaded onto LC-MS. The retention times for the selected four high abundance core KPC peptides were determined with two known KPC-producing isolates (ECNIH2 and ECNIH3) by targeted LC-MS/MS. The collision energy of the chosen precursor was optimized to produce the strongest signals. The isolation width for MS/MS was 4 Da. Targeted LC-MS/MS assay using Q-TOF was run without labeled peptides. In the final targeted LC-MS/MS assay, the retention window was set to 1 min. Spectral acquisition time was set to 300 ms for all peptides. A Med Molecule Column Test Mix Ang 5 (MiChrom Bioresources) was run daily to check column’s performance. To minimize run-to-run carryover, an 18-min protocol with two cycles of column washing/regeneration was performed between samples. In addition, a 60 min column washing/regeneration was run after every 15-sample run to clean the column. Skyline 3.5 software package (MacCross lab) was used for quantitative and relative spectral intensity comparisons.

### Core peptides of KPC variants

The protein sequences of 15 KPC variants (KPC2 to KPC15 and KPC25) were downloaded (http://www.ncbi.nlm.nih.gov/protein Accessed March 1, 2016). The core (common) peptides for KPC were identified with protein sequence alignment tool of MultAlin (http://multalin.toulouse.inra.fr/multalin/multalin.html) and with the web tool Unipept Peptidome Analysis (http://unipept.ugent.be/peptidefinder). In Peptidome Analysis, each protein sequence of 15 KPC variants were uploaded to Unique Peptide Finder as individual FASTA file to obtain a list of core (common) peptides for KPC variants (See supplementary Material, Note 2). We then applied the requirement that each core peptide was present in 14 out of 15 of the sequences. Manual examination of sequence alignment of the 15 KPC variants was used to confirm the identification of core peptides for KPC variants.

## Electronic supplementary material

Supplementary Material

## References

[1] Centers for Disease Control. Antibiotic Resistance Threats in the United States. http://www.cdc.gov/drugresistance/threat-report-2013/ (2013).

[2] World Health Organization. Antimicrobial Resistance: Global Report on Surveillance http://www.who.int/drugresistance/documents/surveillancereport/en/ (2014).

[3] Snitkin ES (2012). Tracking a hospital outbreak of carbapenem-resistant Klebsiella pneumoniae with whole-genome sequencing. Sci Transl Med.

[4] Conlan S (2014). Single-molecule sequencing to track plasmid diversity of hospital-associated carbapenemase-producing Enterobacteriaceae. Sci Transl Med.

[5] Jayol, A., Poirel, L., Dortet, L. & Nordmann, P. National survey of colistin resistance among carbapenemase-producing Enterobacteriaceae and outbreak caused by colistin-resistant OXA-48-producing Klebsiella pneumoniae, France, 2014. *Euro Surveill* **21**, doi:10.2807/1560-7917.ES.2016.21.37.30339 (2016). 10.2807/1560-7917.ES.2016.21.37.30339 PMC5032854 27685838

[6] Nordmann P, Poirel L (2014). The difficult-to-control spread of carbapenemase producers among Enterobacteriaceae worldwide. Clin Microbiol Infect.

[7] Kazmierczak KM (2016). Global Dissemination of blaKPC into Bacterial Species beyond Klebsiella pneumoniae and *In Vitro* Susceptibility to Ceftazidime-Avibactam and Aztreonam-Avibactam. Antimicrob Agents Chemother.

[8] Chavda KD, Chen L, Jacobs MR, Bonomo RA, Kreiswirth BN (2016). Molecular Diversity and Plasmid Analysis of KPC-Producing Escherichia coli. Antimicrob Agents Chemother.

[9] van Duin, D. & Doi, Y. The global epidemiology of carbapenemase-producing Enterobacteriaceae. *Virulence*, 1–10, doi:10.1080/21505594.2016.1222343 (2016). 10.1080/21505594.2016.1222343 PMC5477705 27593176

[10] White House National Action Plan for Combating Antibiotic-Resistant Bacteria. https://www.whitehouse.gov/sites/default/files/docs/national_action_plan_for_combating_antibotic-resistant_bacteria.pdf. (2015).

[11] Monteferrante CG (2016). Evaluation of different pretreatment protocols to detect accurately clinical carbapenemase-producing Enterobacteriaceae by MALDI-TOF. J Antimicrob Chemother.

[12] Ramos AC (2016). Influence of Culture Media on Detection of Carbapenem Hydrolysis by Matrix-Assisted Laser Desorption Ionization-Time of Flight Mass Spectrometry. J Clin Microbiol.

[13] Ghebremedhin B, Halstenbach A, Smiljanic M, Kaase M, Ahmad-Nejad P (2016). MALDI-TOF MS based carbapenemase detection from culture isolates and from positive blood culture vials. Ann Clin Microbiol Antimicrob.

[14] Mirande C (2015). Rapid detection of carbapenemase activity: benefits and weaknesses of MALDI-TOF MS. Eur J Clin Microbiol Infect Dis.

[15] Lasserre C (2015). Efficient Detection of Carbapenemase Activity in Enterobacteriaceae by Matrix-Assisted Laser Desorption Ionization-Time of Flight Mass Spectrometry in Less Than 30 Minutes. J Clin Microbiol.

[16] Sauget M, Cabrolier N, Manzoni M, Bertrand X, Hocquet D (2014). Rapid, sensitive and specific detection of OXA-48-like-producing Enterobacteriaceae by matrix-assisted laser desorption/ionization time-of-flight mass spectrometry. J Microbiol Methods.

[17] Peaper DR (2013). Rapid detection of carbapenemase activity through monitoring ertapenem hydrolysis in Enterobacteriaceae with LC-MS/MS. Bioanalysis.

[18] Hrabak J (2012). Detection of NDM-1, VIM-1, KPC, OXA-48, and OXA-162 carbapenemases by matrix-assisted laser desorption ionization-time of flight mass spectrometry. J Clin Microbiol.

[19] Huber CA (2016). Detection of carbapenemase activity in Enterobacteriaceae using LC-MS/MS in comparison with the neo-rapid CARB kit using direct visual assessment and colorimetry. J Microbiol Methods.

[20] Kulkarni MV (2014). Use of imipenem to detect KPC, NDM, OXA, IMP, and VIM carbapenemase activity from gram-negative rods in 75 minutes using liquid chromatography-tandem mass spectrometry. J Clin Microbiol.

[21] Foschi C (2015). Use of liquid chromatography-tandem mass spectrometry (LC-MS/MS) to detect carbapenemase production in Enterobacteriaceae by a rapid meropenem degradation assay. New Microbiol.

[22] Lau AF (2014). A rapid matrix-assisted laser desorption ionization-time of flight mass spectrometry-based method for single-plasmid tracking in an outbreak of carbapenem-resistant Enterobacteriaceae. J Clin Microbiol.

[23] Youn JH (2016). Clinical Performance of a Matrix-Assisted Laser Desorption Ionization-Time of Flight Mass Spectrometry Method for Detection of Certain blaKPC-Containing Plasmids. J Clin Microbiol.

[24] Charretier, Y. *et al*. Method of detecting at least one mechanism of resistance to carbapenems by mass spectrometry. Patent US 20150031063 (2012).

[25] Charretier Y, Schrenzel J (2016). Mass spectrometry methods for predicting antibiotic resistance. Proteomics Clin Appl.

[26] Chang CJ (2013). Diagnosis of beta-lactam resistance in Acinetobacter baumannii using shotgun proteomics and LC-nano-electrospray ionization ion trap mass spectrometry. Anal Chem.

[27] Fleurbaaij F (2014). Capillary-electrophoresis mass spectrometry for the detection of carbapenemases in (multi-)drug-resistant Gram-negative bacteria. Anal Chem.

[28] Charretier Y (2015). Rapid Bacterial Identification, Resistance, Virulence and Type Profiling using Selected Reaction Monitoring Mass Spectrometry. Sci Rep.

[29] Wang H (2016). A Novel Peptidomic Approach to Strain Typing of Clinical Acinetobacter baumannii Isolates Using Mass Spectrometry. Clin Chem.

[30] Fusaro VA, Mani DR, Mesirov JP, Carr SA (2009). Prediction of high-responding peptides for targeted protein assays by mass spectrometry. Nat Biotechnol.

[31] Glatter T (2012). Large-scale quantitative assessment of different in-solution protein digestion protocols reveals superior cleavage efficiency of tandem Lys-C/trypsin proteolysis over trypsin digestion. J Proteome Res.

[32] Damm M (2012). Can electromagnetic fields influence the structure and enzymatic digest of proteins? A critical evaluation of microwave-assisted proteomics protocols. J Proteomics.

[33] Kim JS, Monroe ME, Camp DG, Smith RD, Qian WJ (2013). In-source fragmentation and the sources of partially tryptic peptides in shotgun proteomics. J Proteome Res.

[34] Centers for Disease Control. Multiplex Real-Time PCR Detection of Klebsiella pneumoniae Carbapenemase (KPC) and New Delhi metallo-beta-lactamase (NDM-1) https://www.cdc.gov/HAI/pdfs/labSettings/KPC-NDM-protocol-2011.pdf (2011).

