# Supplementary material for: Peptide Markers for Rapid Detection of KPC Carbapenemase by LC-MS/MS
Source: Sci Rep. 2017 May 31;7:2531. doi: 10.1038/s41598-017-02749-2 (PMC5451396; doi:10.1038/s41598-017-02749-2)
Supplement: Supplementary file 1 — Supplementary Material [file 41598_2017_2749_MOESM1_ESM.pdf]

Peptide Markers for Rapid Detection of KPC Carbapenemase by LC-MS/MS

Honghui Wang, Steven K. Drake, Jung-Ho Youn, Avi Z. Rosenberg, Yong Chen, Marjan Gucek, Anthony F. Suffredini, John P. Dekker

Supplementary Material

Supplementary Table 1. Peptides for KPC variants and detected spectral numbers by Orbitrap Fusion LC-MS/MS

Mass[a]	Position	Peptide sequence (>=6 or <25 AA in length)	Variant position[b] and/or lack of specificity	ECNIH2	ECNIH3	KPNIH1	KPNIH10
1340.72	99-110	NALVPWSPISEK	KPC4,5,10,11,15: 103P-->R or L	NE	NE	NE	NE
1812.91	164-179	WELELELNSAIPGDAR	KPC12: 168L-->M; KPC25: 169E, 170L	NE	NE	NE	NE
2188.01	236-256	TGTCGVYGTANDYAVVWPTGR	KPC4,6,8,9,15:241V -->G or A; KPC14: del 243-244	NE	NE	NE	NE
1095.59	274-284	HSEAVIAAAAR	KPC3,7,8,10,13,15: 274H-->Y	NE	NE	NE	NE
896.44	153-160	SIGDTTFR	Not specific to KPC	1	1(1)	1	ND
875.48	186-193	AVTESLQK	Not specific to KPC	(1)	ND	ND	(1)
928.45	65-72	FPLCSSFK		ND	ND	ND	ND
1426.76	83-95	SQQQAGLLDTPIR	KPC13: 91D-->G	2 (3)	1 (2)	2 (3)	2 (3)
1319.67	140-152	ELGGPAGLTAFMR		1(2[c])	1(1[c])	2(2[c])	1(2[c])
662.31	180-185	DTSSPR		ND	ND	ND	ND
1063.55	206-213	QQFVDWLK		ND	1(2)	(1)	(1)
856.40	214-221	GNTTGNHR		ND	ND	ND	ND
1199.60	224-235	AAVPADWAVGDK		ND	1	2	2
988.59	73-82	GFLAAAVLAR		3	2	3	2
1197.69	194-205	LTLGSALAAPQR		3	2	2	3
1102.66	257-266	APIVLAVYTR		4	2	2	3
1070.58	285-295	LALEGLGVNGQ	KPC9: missing 292-295	2	3	3	3

Numbers in parentheses correspond to cases where peptide was present in the detected sequence with one missed-cleavage due to incomplete digestion. Red indicates non-core peptides; the remaining 13 peptides meet core criteria. Blue indicates core peptides not specific to KPC. Grey peptides were selected for further workup based on LC-MS/MS performance data, and green were rejected. NE = Not Examined; ND = Not Detected
[a]Average Neutral Mass. [b]Numbering scheme based on sequence of KPC25. [c]Oxidation of methionine present.

Supplementary Table 2. Protein identification by Orbitrap Fusion mass spectrometer

Isolate	Total Proteins	Total peptides	Core & Specific KPC Peptides
ECNIH2	739	2812	6
ECNIH3	941	3741	8
KPNIH1	693	2477	7
KPNIH10	659	2573	7

Settings: Minimum peptides = 2, protein threshold = 1% FDR, peptide threshold = 2% FDR. ECNIH2 and ECNIH10 - *Enterobacter cloacae*. KPNIH1 and KPNIH10 - *Klebsiella pneumoniae*.

Supplementary Table 3. Average intensities and percent change for three peptide peaks in daily positive controls

Day	Peptide	GFLAAAVLAR	LTLGSALAAPQR	APIVLAVYTR
Day 1	Avg intensity	513832	2975468	2176989
	% change over day[a]	-0.4	2.0	-8.1
Day 2	Avg intensity	402908	2633449	1960389
	% change over day	12.5	14.4	0.9
Day 3	Avg intensity	506280	3452186	2344396
	% change over day	-6.3	2.2	-4.2
Day 4	Avg intensity	386975	2333300	1643544
	%CV	11.1	12.5	-4.2
Day 5	Avg intensity	605171	2680534	1936007
	% change over day	-18.4	-11.6	-26.4
Day 6	Avg intensity	915871	2785980	2487575
	% change over day	74.7	-14.3	15.7
Day 7	Avg intensity	491817	2264550	1598329
	% change over day	-11.8	-10.7	-23.3
Day 8	Avg intensity	571352	2880678	1943506
	% change over day	7.1	-2.4	-8.8
Day 9[b]	Avg intensity	267570	740663	689609
	% change over day	0.2	1.3	5.1
Day 10[b]	Avg intensity	179643	883797	489699
	% change over day	2.5	-11.8	-10.6

[a]The % change over day (20 hr period) was calculated as 100*((second control-first control)/first control). [b]Avg intensity drop in Day 9 and 10 was determined to be due to

altered performance of the collision cell. This did not impact identification of the KPC-positive isolates in our clinical samples and the analysis was not repeated.

Supplementary Table 4. Dynamic range and sensitivity for three KPC peptide markers

Sample	Volume KPNIH1 (μl)	Volume matrix (μl)	Dilution factor	Theoretical Ratio (To QC)	GFLAAAVLAR measured ratio	LTLGSALAAPQR measured ratio	APIVLAVYTR measured ratio
1	0	20	N/A	0	- (0.01)	-(0.003)	-(0.01)
2	0.1	19.9	200	0.01	- (0.02)	-(0.01)	-(0.02)
3	0.25	19.75	80	0.025	- (0.04)	+(0.02)	+(0.03)
4	0.5	19.5	40	0.05	+ (0.04)	+(0.03)	+(0.04)
5	1	19	20	0.1	+ (0.13)	+(0.09)	+(0.1)
6	2.5	17.5	8	0.25	+ (0.22)	+(0.17)	+(0.19)
7	5	15	4	0.5	+ (0.49)	+(0.43)	+(0.44)
8[a]	10	10	2	1	+ (1.0)	+(1.0)	+(1.0)
9	20	0	1	2	+ (1.9)	+(1.59)	+(1.78)

+ indicates that the transition rank orders were identified. – indicates that the transition rank orders were not detected or detected with low signals. [a]Measured intensity ratio was normalized to sample 8 which has dilution factor of 2, equivalent to daily positive control.

Supplementary Figure 1.

```
           1       10        20        30        40        50        60        70        80        90        100       110       120       130
           |--------+---------+---------+---------+---------+---------+---------+---------+---------+---------+---------+---------+---------|
    KPC-3  MSLYRRLVLLSCLSWPLAGFSATALTNLVAEPFAKLEQDFGGSIGVYAMDTGSGATVSYRAEERFPLCSSFKGFLAAAVLARSQQQAGLLDTPIRYGKNALVPWSPISEKYLTTGMTVAELSAAAVQYSD
    KPC-7  MSLYRRLVLLSCLSWPLAGFSATALTNLVAEPFAKLEQDFGGSIGVYAIDTGSGATVSYRAEERFPLCSSFKGFLAAAVLARSQQQAGLLDTPIRYGKNALVPWSPISEKYLTTGMTVAELSAAAVQYSD
   KPC-12  MSLYRRLVLLSCLSWPLAGFSATALTNLVAEPFAKLEQDFGGSIGVYAMDTGSGATVSYRAEERFPLCSSFKGFLAAAVLARSQQQAGLLDTPIRYGKNALVPWSPISEKYLTTGMTVAELSAAAVQYSD
    KPC-2  MSLYRRLVLLSCLSWPLAGFSATALTNLVAEPFAKLEQDFGGSIGVYAMDTGSGATVSYRAEERFPLCSSFKGFLAAAVLARSQQQAGLLDTPIRYGKNALVPWSPISEKYLTTGMTVAELSAAAVQYSD
    KPC-6  MSLYRRLVLLSCLSWPLAGFSATALTNLVAEPFAKLEQDFGGSIGVYAMDTGSGATVSYRAEERFPLCSSFKGFLAAAVLARSQQQAGLLDTPIRYGKNALVPWSPISEKYLTTGMTVAELSAAAVQYSD
    KPC-8  MSLYRRLVLLSCLSWPLAGFSATALTNLVAEPFAKLEQDFGGSIGVYAMDTGSGATVSYRAEERFPLCSSFKGFLAAAVLARSQQQAGLLDTPIRYGKNALVPWSPISEKYLTTGMTVAELSAAAVQYSD
   KPC-13  MSLYRRLVLLSCLSWPLAGFSATALTNLVAEPFAKLEQDFGGSIGVYAMDTGSGATVSYRAEERFPLCSSFKGFLAAAVLARSQQQAGLLGTPIRYGKNALVPWSPISEKYLTTGMTVAELSAAAVQYSD
    KPC-5  MSLYRRLVLLSCLSWPLAGFSATALTNLVAEPFAKLEQDFGGSIGVYAMDTGSGATVSYRAEERFPLCSSFKGFLAAAVLARSQQQAGLLDTPIRYGKNALVRWSPISEKYLTTGMTVAELSAAAVQYSD
   KPC-10  MSLYRRLVLLSCLSWPLAGFSATALTNLVAEPFAKLEQDFGGSIGVYAMDTGSGATVSYRAEERFPLCSSFKGFLAAAVLARSQQQAGLLDTPIRYGKNALVRWSPISEKYLTTGMTVAELSAAAVQYSD
   KPC-11  MSLYRRLVLLSCLSWPLAGFSATALTNLVAEPFAKLEQDFGGSIGVYAMDTGSGATVSYRAEERFPLCSSFKGFLAAAVLARSQQQAGLLDTPIRYGKNALVLWSPISEKYLTTGMTVAELSAAAVQYSD
   KPC-25  MSLYRRLVLLSCLSWPLAGFSATALTNLVAEPFAKLEQDFGGSIGVYAMDTGSGATVSYRAEERFPLCSSFKGFLAAAVLARSQQQAGLLDTPIRYGKNALVPWSPISEKYLTTGMTVAELSAAAVQYSD
    KPC-4   SLYRRLVLLSCLSWPLAGFSATALTNLVAEPFAKLEQDFGGSIGVYAMDTGSGATVSYRAEERFPLCSSFKGFLAAAVLARSQQQAGLLDTPIRYGKNALVRWSPISEKYLTTGMTVAELSAAAVQYSD
   KPC-15  MSLYRRLVLLSCLSWPLAGFSATALTNLVAEPFAKLEQDFGGSIGVYAMDTGSGATVSYRAEERFPLCSSFKGFLAAAVLARSQQQAGLLDTPIRYGKNALVRWSPISEKYLTTGMTVLELSAAAVQYSD
   KPC-14  MSLYRRLVLLSCLSWPLAGFSATALTNLVAEPFAKLEQDFGGSIGVYAMDTGSGATVSYRAEERFPLCSSFKGFLAAAVLARSQQQAGLLDTPIRYGKNALVPWSPISEKYLTTGMTVAELSAAAVQYSD
    KPC-9       RLVLLSCLSWPLAGFSATALTNLVAEPFAKLEQDFGGSIGVYAMDTGSGATVSYRAEERFPLCSSFKGFLAAAVLARSQQQAGLLDTPIRYGKNALVPWSPISEKYLTTGMTVAELSAAAVQYSD
Consensus  mslyrRLVLLSCLSWPLAGFSATALTNLVAEPFAKLEQDFGGSIGVYAMDTGSGATVSYRAEERFPLCSSFKGFLAAAVLARSQQQAGLLDTPIRYGKNALVpWSPISEKYLTTGMTVAELSAAAVQYSD

           131     140       150       160       170       180       190       200       210       220       230       240       250       260
           |--------+---------+---------+---------+---------+---------+---------+---------+---------+---------+---------+---------+---------|
    KPC-3  NAAANLLLKELGGPAGLTAFMRSIGDTTFRLDRWELEL--NSAIPGDARDTSSPRAVTESLQKLTLGSALAAPQRQQFVDWLKGNTTGNHRIRAAVPADWAVGDKTGTCGVYGTANDYAVVWPTGRAPIV
    KPC-7  NAAANLLLKELGGPAGLTAFMRSIGDTTFRLDRWELEL--NSAIPGDARDTSSPRAVTESLQKLTLGSALAAPQRQQFVDWLKGNTTGNHRIRAAVPADWAVGDKTGTCGVYGTANDYAVVWPTGRAPIV
   KPC-12  NAAANLLLKELGGPAGLTAFMRSIGDTTFRLDRWELEM--NSAIPGDARDTSSPRAVTESLQKLTLGSALAAPQRQQFVDWLKGNTTGNHRIRAAVPADWAVGDKTGTCGVYGTANDYAVVWPTGRAPIV
    KPC-2  NAAANLLLKELGGPAGLTAFMRSIGDTTFRLDRWELEL--NSAIPGDARDTSSPRAVTESLQKLTLGSALAAPQRQQFVDWLKGNTTGNHRIRAAVPADWAVGDKTGTCGVYGTANDYAVVWPTGRAPIV
    KPC-6  NAAANLLLKELGGPAGLTAFMRSIGDTTFRLDRWELEL--NSAIPGDARDTSSPRAVTESLQKLTLGSALAAPQRQQFVDWLKGNTTGNHRIRAAVPADWAVGDKTGTCGGYGTANDYAVVWPTGRAPIV
    KPC-8  NAAANLLLKELGGPAGLTAFMRSIGDTTFRLDRWELEL--NSAIPGDARDTSSPRAVTESLQKLTLGSALAAPQRQQFVDWLKGNTTGNHRIRAAVPADWAVGDKTGTCGGYGTANDYAVVWPTGRAPIV
   KPC-13  NAAANLLLKELGGPAGLTAFMRSIGDTTFRLDRWELEL--NSAIPGDARDTSSPRAVTESLQKLTLGSALAAPQRQQFVDWLKGNTTGNHRIRAAVPADWAVGDKTGTCGVYGTANDYAVVWPTGRAPIV
    KPC-5  NAAANLLLKELGGPAGLTAFMRSIGDTTFRLDRWELEL--NSAIPGDARDTSSPRAVTESLQKLTLGSALAAPQRQQFVDWLKGNTTGNHRIRAAVPADWAVGDKTGTCGVYGTANDYAVVWPTGRAPIV
   KPC-10  NAAANLLLKELGGPAGLTAFMRSIGDTTFRLDRWELEL--NSAIPGDARDTSSPRAVTESLQKLTLGSALAAPQRQQFVDWLKGNTTGNHRIRAAVPADWAVGDKTGTCGVYGTANDYAVVWPTGRAPIV
   KPC-11  NAAANLLLKELGGPAGLTAFMRSIGDTTFRLDRWELEL--NSAIPGDARDTSSPRAVTESLQKLTLGSALAAPQRQQFVDWLKGNTTGNHRIRAAVPADWAVGDKTGTCGVYGTANDYAVVWPTGRAPIV
   KPC-25  NAAANLLLKELGGPAGLTAFMRSIGDTTFRLDRWELELELNSAIPGDARDTSSPRAVTESLQKLTLGSALAAPQRQQFVDWLKGNTTGNHRIRAAVPADWAVGDKTGTCGVYGTANDYAVVWPTGRAPIV
    KPC-4  NAAANLLLKELGGPAGLTAFMRSIGDTTFRLDRWELEL--NSAIPGDARDTSSPRAVTESLQKLTLGSALAAPQRQQFVDWLKGNTTGNHRIRAAVPADWAVGDKTGTCGGYGTANDYAVVWPTGRAPIV
   KPC-15  NAAANLLLKELGGPAKLTAFMRSIGDTTFRLDRWELEL--NSAIPGDARDTSSPRAVTESLQKLTLGSALAAPQRQQFVDWLKGNTTGNHRIRAAVPADWAVGDKTGTCGGYGTANDYAVVWPTGRAPIV
   KPC-14  NAAANLLLKELGGPAGLTAFMRSIGDTTFRLDRWELEL--NSAIPGDARDTSSPRAVTESLQKLTLGSALAAPQRQQFVDWLKGNTTGNHRIRAAVPADWAVGDKTGTCGVY--ANDYAVVWPTGRAPIV
    KPC-9  NAAANLLLKELGGPAGLTAFMRSIGDTTFRLDRWELEL--NSAIPGDARDTSSPRAVTESLQKLTLGSALAAPQRQQFVDWLKGNTTGNHRIRAAVPADWAVGDKTGTCGAYGTANDYAVVWPTGRAPIV
Consensus  NAAANLLLKELGGPAGLTAFMRSIGDTTFRLDRWELEL  NSAIPGDARDTSSPRAVTESLQKLTLGSALAAPQRQQFVDWLKGNTTGNHRIRAAVPADWAVGDKTGTCGvYgtANDYAVVWPTGRAPIV

           261     270       280       290  295
           |--------+---------+---------+----|
    KPC-3  LAVYTRAPNKDDKYSEAVIAAAARLALEGLGVNGQ
    KPC-7  LAVYTRAPNKDDKYSEAVIAAAARLALEGLGVNGQ
   KPC-12  LAVYTRAPNKDDKHSEAVIAAAARLALEGLGVNGQ
    KPC-2  LAVYTRAPNKDDKHSEAVIAAAARLALEGLGVNGQ
    KPC-6  LAVYTRAPNKDDKHSEAVIAAAARLALEGLGVNGQ
    KPC-8  LAVYTRAPNKDDKYSEAVIAAAARLALEGLGVNGQ
   KPC-13  LAVYTRAPNKDDKYSEAVIAAAARLALEGLGVNGQ
    KPC-5  LAVYTRAPNKDDKHSEAVIAAAARLALEGLGVNGQ
   KPC-10  LAVYTRAPNKDDKYSEAVIAAAARLALEGLGVNGQ
   KPC-11  LAVYTRAPNKDDKHSEAVIAAAARLALEGLGVNGQ
   KPC-25  LAVYTRAPNKDDKHSEAVIAAAARLALEGLGVNGQ
    KPC-4  LAVYTRAPNKDDKHSEAVIAAAARLALEGLGVNGQ
   KPC-15  LAVYTRAPNKDDKYSEAVIAAAARLALEGLGVNGQ
   KPC-14  LAVYTRAPNKDDKHSEAVIAAAARLALEGLGVNGQ
    KPC-9  LAVYTRAPNKDDKYSEAVIAAAARLALEGLG
Consensus  LAVYTRAPNKDDKySEAVIAAAARLALEGLGvngq
```

Multiple sequence alignment constructed for the 15 KPC variants included in this work. Alignment built with MultAlin (http://multalin.toulouse.inra.fr/multalin/).

Supplementary Figure 2.

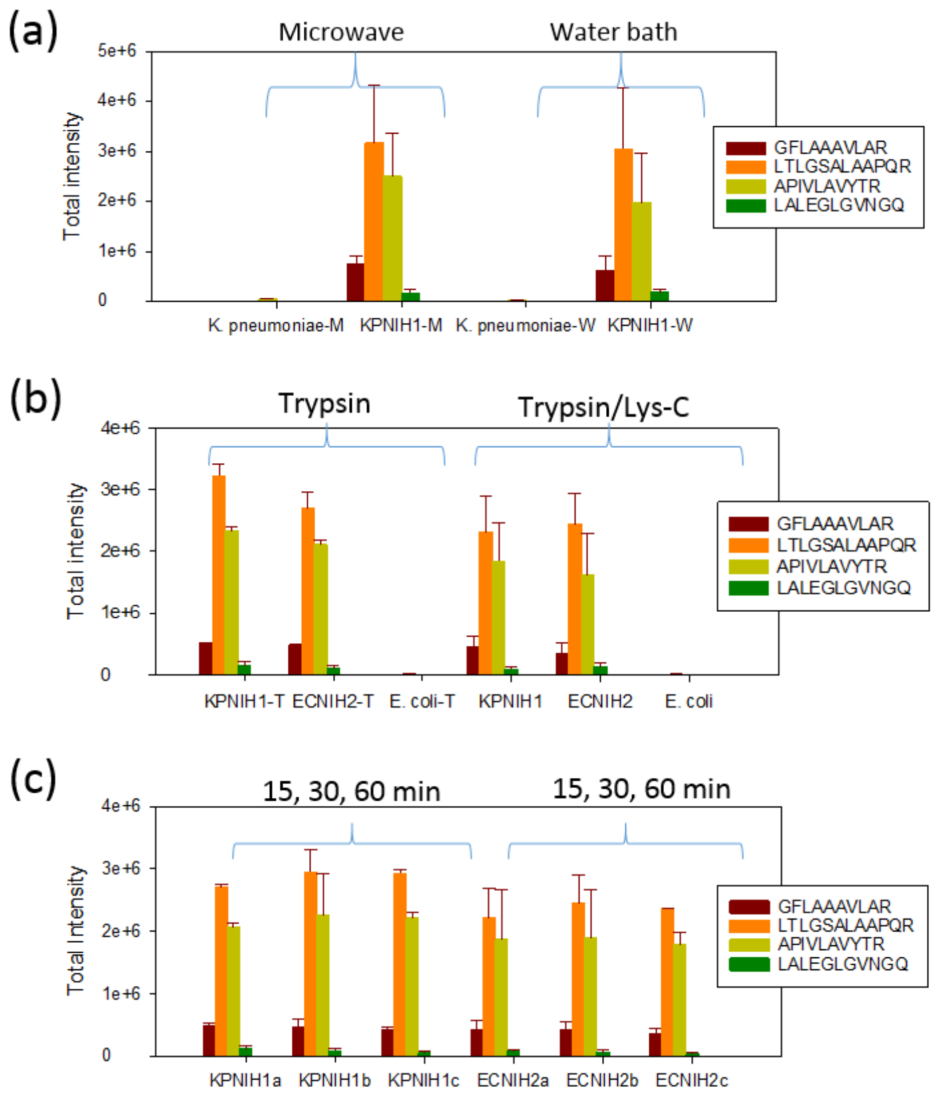

Protein digestion protocol optimization for targeted peptides. Three separate digestions of the same isolate are averaged and presented with standard deviation. (a) Microwave (M) or water bath (W) effect on four KPC peptides with 30 min digestion and 1 µg Trypsin/Lys-C mix. KPNIH1 is a KPC-positive isolate and *K. pneumoniae* is a KPC-negative isolate that was included to study background noise and interfering peaks. (b) Trypsin (T) or Trypsin/Lys-C effect on four KPC peptides with 1 µg enzyme and 30 min digestion in water bath. ECNIH2 is a KPC-positive isolate. *E. coli* is a KPC-negative isolate. (C) Digestion time effect on four KPC peptides in water bath with 1 µg Trypsin for two different KPC-positive isolates. Note: in (a) and (b), vortex resuspensions were carried out at room temperature for 10 minutes.

Supplementary Figure 3.

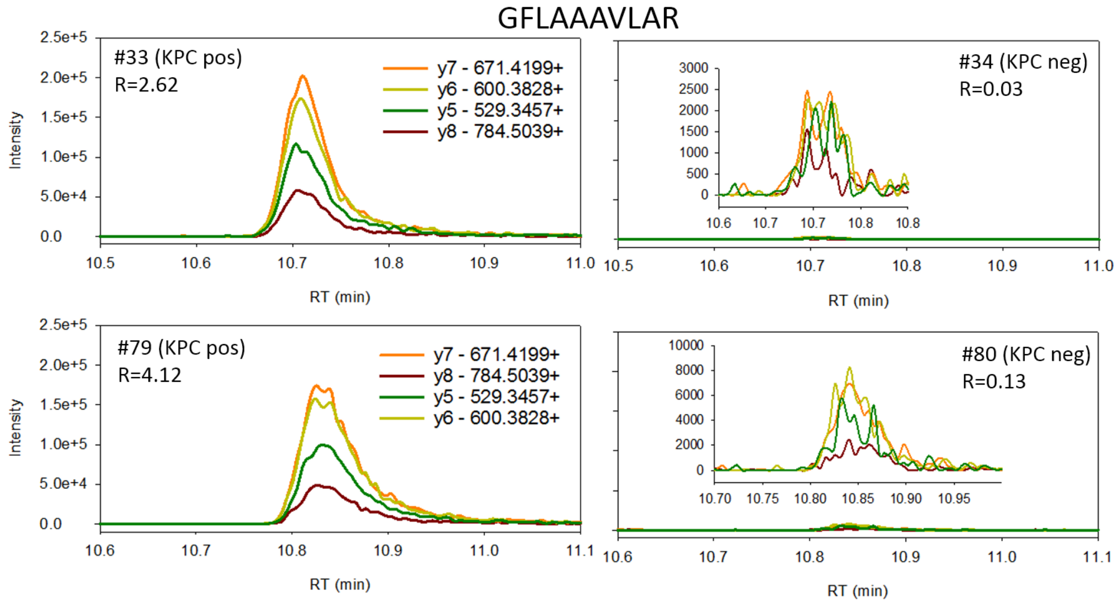

Two examples of carryover of GFLAAAVLAR from a positive isolate with high signal intensity to the next consecutive negative specimen. Top panel: KPC-positive isolate #33 followed by KPC-negative isolate #34. Bottom panel: KPC-positive isolate #79 followed by KPC-negative isolate #80. In both cases, carryover effect is noted, and was ruled carryover by the expert rules explained in main text. Additionally, independent repeat testing (performed separately from validation) on sample #80 confirmed the interpretation of signal as carryover (data not shown). R is intensity ratio normalized to daily positive QC control. Note that horizontal scale is same between left and right panels. Magnified inset is included in right panels for detail.

Supplementary Figure 4.

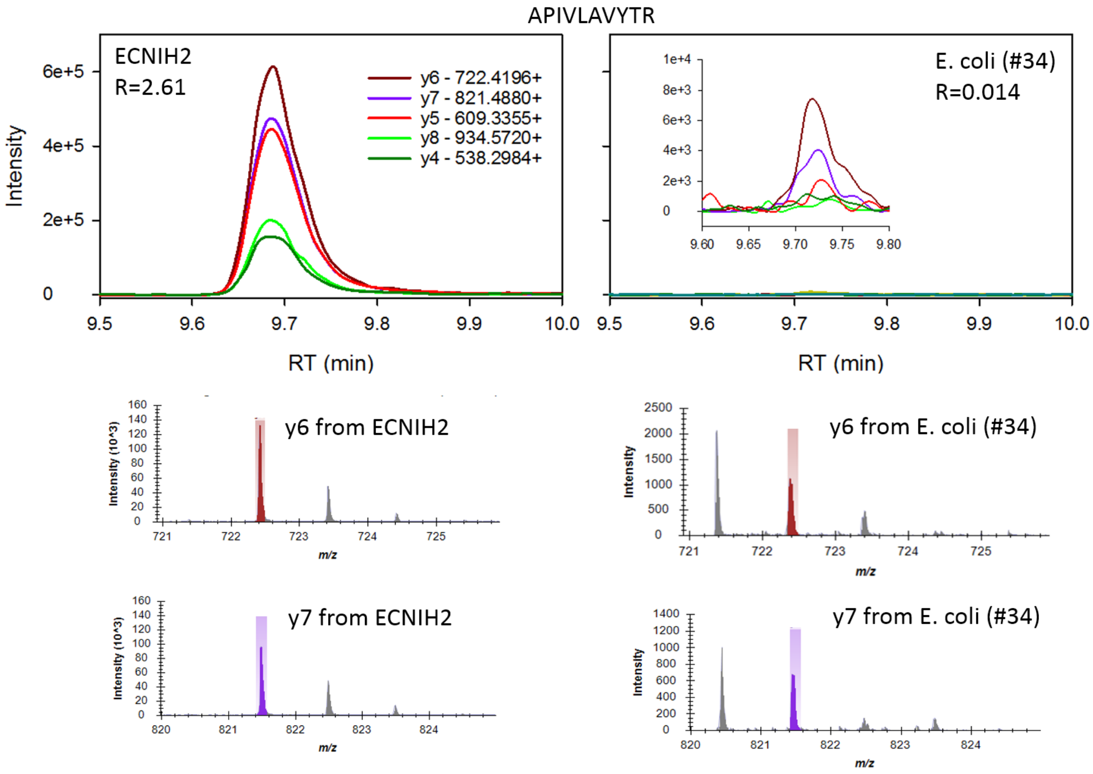

Top panel: Chromatograms demonstrated similar transition rank orders in ECNIH2 (KPC-positive) and *E. coli* (#34) (KPC-negative) for peptide marker APIVLAVYTR. The peak in *E. coli* (#34) was confirmed as interfering by its MS spectra. The spectra of y6 and y7 in ECNIH2 matched to their monoisotopic m/z values (left-bottom two panels), while the spectra of y6 and y7 in E. coli (#34) did not match to their monoisotopic m/z values (right-bottom two panels). R is intensity ratio normalized to the daily positive control.

Supplementary Figure 5.

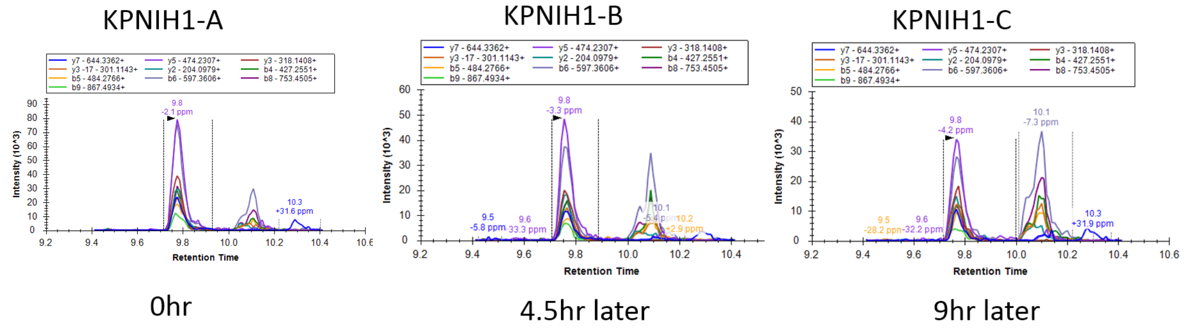

Deamidation of LALEGLGVNGQ peptide. Two peaks were observed at retention times of 9.8 min and 10.1 min. This peptide contained N and Q amino acids which can undergo deamidation. The samples were placed on autosampler at room temperature and sampled at different time points. KPNIH1-A,B,C were separate preparations from the same lysate. LALEGLGVNGQ was not included in the validation set due to this observed deamidation.

Supplementary Figure 6.

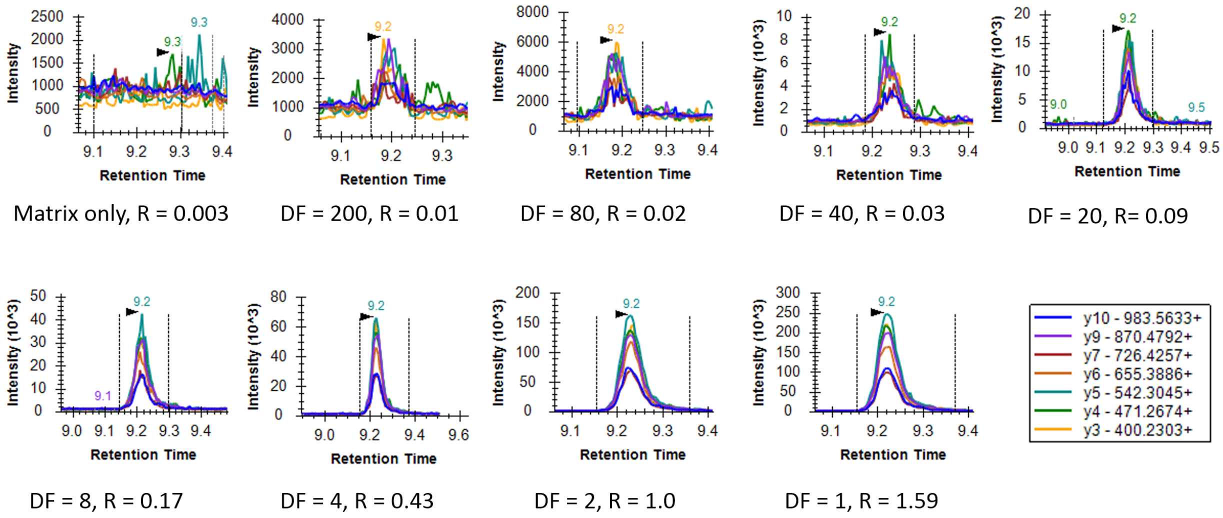

LC-MS chromatogram of peptide LTLGSALAAPQR through dilution series demonstrating dynamic range. The lysate of KPNIH1 (KPC-positive) was diluted with the lysate of K. pneumonia (KPC-negative). DF is dilution factor. DF = 2 is equivalent to the daily positive control and was used for R values. R is intensity averaged from 2 measurements.

Supplementary Note 1. Deamidation of LALEGLGVNGQ peptide.
While most of the studied peptides were found to be stable, deamidation of the LALEGLGVNGQ peptide was observed during the test run (Supplementary Figure 5). Two peaks were observed at retention times of 9.8 min and 10.1 min. This peptide contained amino acid groups of N and Q which can undergo deamidation. Separated LC-MS/MS confirmed the presence of NQ deamidation (data not shown). Deamidation of this peptide increased its molecular weight by 0.98 Da, which was still within the isolation width of 4 Da in the targeted LC-MS/MS method. N and Q were at the C-terminus, and the deamidation resulted in the m/z change in y-ions and had no effect on the m/z for most of b-ions. Thus a deamidated peptide with similar b-ion transition profiling (RT 10.1 min) to that of non-deamidated peptide (RT 9.8 min) was detected. This peptide may not be a good candidate as a peptide marker for the KPC protein. However, if we considered only the b-ions and integrated the two peaks together, it still yielded reasonable reproducibility (data not shown). Refrigeration to 4°C was shown to reduce the rate of deamination (data not shown). This peptide was not used for method validation due to deamidation.

Supplementary Note 2. Uploading FASTA files to (http://unipept.ugent.be/peptidefinder).
When uploading a single KPC protein sequence as single FASTA file to Unique Peptide Finder, a ">" sign was added as new line at the end of the KPC sequence. Without this, Unique Peptide Finder was unable to read last line of the protein sequence for possible program bug (accessed March-April 2016).
